# Engaging with digital health: a cross-sectional study of older adults’ use of online health information exchange

**DOI:** 10.3389/fdgth.2026.1937500

**Published:** 2026-10-07

**Authors:** Samin Sepahniya, Yves Bachofner, Lucy Bayer-Oglesby, Alexander Seifert, Carlo Fabian

**Affiliations:** 1 Institute for Social Work and Health, School of Social Work, University of Applied Sciences and Arts Northwestern Switzerland, Olten, Switzerland; 2 Institute for Integration and Participation, School of Social Work, University of Applied Sciences and Arts Northwestern Switzerland, Olten, Switzerland

**Keywords:** digital competence, health literacy, healthy aging, older adults, online health information exchange

## Abstract

**Background:**

Online health information exchange (OHIE) increasingly shapes how individuals interact with healthcare providers and peers. Yet, despite older adults’ often high need for health information, their engagement with OHIE remains insufficiently understood, given persistent age-related differences in digital access and skills.

**Methods:**

This study draws on a population-based survey of older adults aged 60 + in Switzerland (*N* = 1,261) to analyze three types of OHIE: interactions with doctors, other healthcare providers, and peers. Logistic regression models were used to assess how sociodemographic, health-related, behavioral, and competence-based factors are associated with OHIE.

**Results:**

Older adults reported higher use of digital interactions with professionals than with peers. Digital competence was significantly associated with OHIE with doctors and other healthcare providers across all lower competence levels, and, to a more limited extent, with peer-based OHIE, where the association was significant only for basic competence. Peer-based OHIE was predicted by lower levels of education and a greater tendency to seek social support. Common barriers among non-users included concerns about credibility and distrust, as well as a lack of digital experience.

**Conclusions:**

OHIE among older adults reflects differentiated patterns depending on the interaction context. While OHIE with professionals is primarily associated with digital competence, peer-based digital interactions appear to be shaped more by sociodemographic (such as education) and motivational aspects (such as support seeking). These findings highlight the importance of addressing not only digital skills but also trust, usability, and digital judgment when designing inclusive digital health environments for aging populations.

## 1 Introduction

### 1.1 Background

Online health information exchange (OHIE) has become an increasingly important part of how individuals access, interpret, and discuss health-related information (1). Online patient portals, telemedicine services, health websites, social media, and peer-based digital communities have expanded the opportunities for health-related exchange with peers and health care professionals (2, 3). These developments are particularly relevant for older adults, who are more likely to be managing chronic conditions, navigating complex care needs, and requiring regular access to health information and support (4). However, the growing digitalization of health communication also introduces new demands that may disproportionately challenge older populations, including navigating unfamiliar interfaces, assessing the credibility of digital information, and deciding when and how to engage in digital health exchange (5, 6).

Older adults are increasingly engaging with digital health resources, yet their participation in OHIE remains shaped by inequalities in access, skills, and confidence (4). Although digital health tools can improve access to information, support self-management, and facilitate communication with peers and health care professionals (2, 3), older adults often face age-specific barriers to the use of such tools (4, 7, 8). These include lower digital competence; limited confidence in using digital technologies; concerns regarding privacy and misinformation; and age-related cognitive, sensory, or functional changes that may complicate digital navigation (7–11). Thus, the increasing integration of digital tools into health communication may both expand access to health-related resources and reinforce existing inequalities in later life (12, 13).

Previous research has revealed that older adults increasingly use digital media to search for health information, communicate with health care professionals, and seek support from others with similar health concerns (8, 10, 11, 14). However, much of this literature has examined digital health engagement broadly, often focusing on general online health information seeking, eHealth literacy, or technology adoption. Consequently, less is known about whether the predictors of digital health engagement differ by interaction target (15). In particular, prior studies have rarely distinguished between OHIE with doctors, other health care providers, and peers, although these forms of exchange may involve different motivations, expectations, and barriers. Such distinctions are important because professional and peer-based OHIE may depend on different combinations of individual resources, social motivations, and trust-related considerations.

### 1.2 Framework for digital health equity

To better understand the differences in the use of OHIE with doctors, other health care providers, and peers, as well as the factors explaining these differences, this study draws on Richardson et al.'s framework for digital health equity (16). This framework conceptualizes digital health engagement as shaped by interacting determinants across four levels: individual, interpersonal, community, and societal. At the individual level, engagement depends on personal resources, such as education, health literacy, and digital competence. At the interpersonal level, it is shaped by social support structures and help-seeking orientations. At the community level, digital engagement is influenced by local opportunity structures, such as geographical location and access to health care services. At the societal level, engagement is embedded in broader institutional and normative conditions, such as trust in digital systems and the perceived legitimacy of online health resources. Applying this framework enables us to conceptualize OHIE as a socially and structurally embedded health behavior shaped by trust, context, resources, and relationships. In this study, the framework is applied heuristically to organize the individual-, interpersonal-, community-, and societal-level factors considered in our analyses, rather than as a fully specified multi-level model whose cross-level mechanisms are empirically tested.

### 1.3 Aims

Against this background, the present study examines how older adults in Switzerland engage in OHIE and which factors are associated with different forms of its use. Drawing on Richardson et al.'s framework for digital health equity (16), we conceptualize OHIE as a digitally mediated health behavior shaped by determinants at multiple levels, including individual resources, interpersonal orientations, community-level conditions, and broader trust- and credibility-related concerns. Using a sample of adults aged 60 years and older recruited through stratified random sampling in Switzerland, we distinguish between OHIE with doctors, other health care providers, and peers. Based on previous findings (15, 17), we formulated the following hypotheses: (H1) higher digital competence and health literacy are associated with greater engagement in OHIE; (H2) professional forms of OHIE are closely linked to individual and community-level resources; (H3) peer-based OHIE is strongly related to interpersonal factors, such as social support-seeking. In addition, we examine whether trust- and credibility-related concerns constitute key barriers among older adults who do not engage in OHIE.

## 2 Methods

### 2.1 Study design and participants

This study is based on a cross-sectional survey design. The target population comprised individuals aged 60 years and older residing in private households throughout Switzerland. A stratified random sampling approach was used to ensure demographic and regional representativeness. Address data were obtained from the Swiss Federal Statistical Office (SRPH) and supplemented with additional addresses from the commercial provider AZ Direct, as high response rates expected for SRPH-based sampling are no longer typically achieved in social research (18). This supplementation ensured an adequate sample size while maintaining data quality. Data collection was conducted by the external Swiss research institute Demo Scope AG between June 27 and August 20, 2024.

A total of 8,311 individuals received a mailed invitation to participate. Of these, 74 invitations were undeliverable (e.g., invalid address), resulting in a net sample of 8,237. In total, 1,367 respondents completed the survey, corresponding to a net response rate of 16.6%. Documented reasons for nonresponse included refusals (*n* = 77), withdrawals due to health-related or other reasons (*n* = 23), and language barriers (*n* = 3); remaining non-participation reflected non-contact or nonresponse after invitation. Participants could choose between an online format (CAWI, *n* = 1,237) and a paper-based version (PAPI, *n* = 130), with the questionnaire available in German, French, and Italian, the official languages of Switzerland. After applying thorough data cleaning procedures to remove incomplete responses (also excluding questionnaires with very short completion times and more than 95% “no answer” responses, resulting in the removal of 42 cases), 1,325 valid cases remained. For this analysis, we included only respondents who were internet users (*N* = 1,261; 95.2%).

The Ethics Committee Northwest and Central Switzerland (EKNZ; Req-2023-00727) reviewed the study and concluded that it did not fall within the scope of the Human Research Act (HRA Art. 2), thus exempting it from the requirement for formal ethical approval. All participants provided informed consent, and all data were anonymized before analysis.

### 2.2 Measures

Outcome variables. The operationalization of OHIE captures three types of digital health-related interactions. Participants were asked how many days in a typical week they used digital offerings to obtain health-related information through (1) digital interaction with doctors, (2) digital interaction with other healthcare providers, and (3) digital interaction with peers (i.e., individuals with similar health concerns). Response options were: “More than once per day, Once a day, 4-6 days per week, 1-3 days per week, less than once per week, I don't use it, but I’m interested, I don't use it, and I’m not interested in it either.” Each of the three items was recoded separately into a binary outcome variable (OHIE with doctors, OHIE with other healthcare providers, and OHIE with peers). The original frequency distribution was highly skewed toward non-use; we therefore dichotomized the measure to distinguish engagement from non-engagement, consistent with established methods in prior research on Online Health Information–Seeking (19). Participants were classified as users if they reported using any form of interactive digital communication to share, seek, or discuss health-related information with professionals or peers (i.e., more than once per day to less than once per week). Predictor variables. Predictor variables were selected based on prior research on digital health engagement in later life (15) and organized according to the framework for digital health equity (16), which conceptualizes digital health engagement as shaped by determinants at the individual, interpersonal, community, and societal levels.

Individual-level determinants included gender, educational level, financial situation, subjective health status, frequency of internet use, digital competence, and health literacy. Gender was coded as female or male, educational level as compulsory, upper secondary, or tertiary, and financial situation was assessed using an item adapted from the Swiss SILC survey (20) and categorized as “very/rather difficult,” “rather easy,” or “easy/very easy.” Subjective health status was adapted from the Swiss Health Survey (21) and categorized as “very poor to mediocre” or “good to very good.” Frequency of internet use was categorized as “(almost) daily use” versus “less than (almost) daily use,” following previous studies (14). Digital competence was assessed using the DigCompSAT instrument developed by Clifford et al. (22) and adapted by Stürz et al. (23), and categorized into four levels: low, basic, intermediate, and advanced. Health literacy was measured using the validated 12-item short scale HLS19-Q12 and categorized as deficient, problematic, sufficient, or excellent (24). The correlation between digital competence and health literacy was low (Pearson's r = 0.26), allowing both variables to be included in the models without concerns about multicollinearity.

Interpersonal determinants included living arrangements and social support-seeking behavior. Living arrangements were coded as living alone versus living with others. Social support-seeking behavior was assessed using items from the Support Seeking subscale of the Berlin Social Support Scales (BSSS) (25), which captures individuals' tendency to seek support in emotionally or practically challenging situations. Responses were provided on a Likert-type scale and averaged across five items, with higher scores indicating a stronger tendency to actively seek support from others.

Community-level determinants included residence location and healthcare embeddedness. Residence location was categorized as rural, intermediate, or urban and included as an indicator of local opportunity structures. Healthcare embeddedness was operationalized as the number of medical treatments in the past 12 months, excluding dental visits. This measure was adapted from the Swiss Health Survey (21) and dichotomized based on the sample mean into below-average and above-average healthcare use.

Societal-level determinants were not directly included in the regression models but were considered in the descriptive analysis of non-use barriers. In line with the framework for digital health equity (16), these factors capture broader normative and institutional conditions shaping digital health engagement, particularly trust in digital systems, concerns about credibility, and general distrust toward digital health environments. To account for these factors descriptively, respondents who did not engage in any form of OHIE were asked to indicate their reasons for non-use.

Multicollinearity among all predictors in each of the three models was assessed using variance inflation factors (VIF) and tolerance statistics. Across the three models, VIF values ranged from 1.076 to 2.933, while tolerance values ranged from 0.341 to 0.929, indicating no substantial multicollinearity. The correlation between digital competence and health literacy specifically was low (Pearson's r = 0.26).

### 2.3 Analytic strategy

All analyses were conducted using SPSS (version 28). Descriptive statistics were used to compare the characteristics of OHIE users and non-users. Chi^2^ tests assessed significant differences between users and non-users. Separate logistic regression models were conducted for the three OHIE outcomes to identify predictors of different digital interaction contexts. Each model included socio-demographic variables, health-related factors, and behavioral and individual characteristics. Model fit was assessed using the Hosmer-Lemeshow goodness-of-fit test. Participants were excluded from the multivariable models via listwise deletion of cases with missing values on any model predictor. To assess whether this reduction introduced missing-data bias, participants with complete data on all model predictors were compared with those with missing data on at least one predictor, using independent-samples t-tests and chi-square tests for age, sex, and education. No significant differences emerged between the two groups in age [t(1259) = −1.57, *p* = .118], sex [*χ*^2^(1) = 0.01, *p* = .909], or education [*χ*^2^(2) = 2.80, *p* = .246], suggesting that missingness was not strongly related to these characteristics.

## 3 Results

### 3.1 Prevalence and bivariate differences in OHIE

The characteristics of the sample and the prevalence of the three types of OHIE are presented in Table 1. Overall, 36.2% of older adults (*n* = 449) reported using digital services to interact with doctors, 32.2% (*n* = 401) reported digital interactions with other healthcare providers, and 14.0% (*n* = 174) exchanged health-related information online with peers in similar health situations.

**Table 1:** Sociodemographic characteristics and digital health-related characteristics of the study sample (*N* = 1,261).

		OHIE with doctors			OHIE with other healthcare providers			OHIE with peers		
	Sample (*n* = 1,261) *n* (%)	User (*n* = 449) %	Non-User (*n* = 790) %	*p* Chi-Quadrat-Test	User (*n* = 401) %	Non-User (*n* = 846) %	*p* Chi-Quadrat Test	User (*n* = 174) %	Non-User (*n* = 1067) %	*p* Chi-Quadrat Test
Age groups				0.010			0.945			0.059
60-69	666 (52.8)	33.4	66.6		32.0	68.0		12.0	88.0	
70-79	438 (34.7)	37.0	63.0		32.0	68.0		15.4	84.6	
80-100	157 (12.5)	46.4	53.6		33.3	66.7		18.7	81.3	
(registered) Gender				0.018			0.004			0.762
Female	539 (42.7)	32.5	67.5		27.7	72.3		14.4	85.6	
Male	722 (57.3)	39.0	61.0		35.5	64.5		13.8	86.2	
Education^a^				<0.001			0.002			<0.001
Compulsory	70 (5.6)	23.5	76.5		22.4	77.6		30.4	69.6	
Secondary school II	714 (57.5)	31.6	68.4		29.3	70.7		12.4	87.6	
Tertiary level	458 (36.9)	45.7	54.3		38.1	61.9		14.2	85.8	
Financial situation^b^				0.082			0.191			0.170
Very difficult to rather difficult	236 (19.5)	30.7	69.3		28.4	71.6		16.2	83.8	
Rather simple	334 (27.6)	36.9	63.1		35.8	64.2		16.4	83.6	
Easy to very easy	639 (52.9)	39.0	61.0		33.0	67.0		12.5	87.5	
Residence location				0.002			0.367			0.779
Rural	265 (21.0)	27.2	72.8		29.7	70.3		14.9	85.1	
Intermediate	278 (22.0)	40.1	59.9		35.3	64.7		14.8	85.2	
Urban	718 (57.0)	38.1	61.9		31.9	68.1		13.4	86.6	
Living Arrangement^c^				0.002			0.356			0.675
Living alone	292 (23.8)	29.2	70.8		30.1	69.9		13.5	86.5	
Not alone	936 (76.2)	39.1	60.9		33.0	67.0		14.5	85.5	

Sample characteristics and characteristics of OHIE users and non-users across three types of digital interactions: with doctors, other healthcare providers, and peers. The table includes differences and significance tests between users and non-users in each category.

a with 19 missings.

b with 52 missings.

c with 33 missings.

Bivariate analyses revealed differences in OHIE use across several sociodemographic characteristics. OHIE with doctors was significantly more common among older age groups, men, and participants with higher educational attainment than among their respective reference groups. A similar pattern was observed for OHIE with other healthcare providers, with higher use among men than women and among participants with higher education than those with lower education. In contrast, peer-based OHIE showed no significant differences by gender or age. However, differences emerged by educational attainment, with participants with lower levels of education reporting peer-based OHIE more often than those with higher educational attainment.

Additional bivariate differences were found for OHIE with doctors. Urban residents reported digital interactions with doctors more frequently than those living in rural areas, and participants living alone reported lower use than those living with others. None of the remaining characteristics showed statistically significant differences in the bivariate analyses.

### 3.2 Logistic regression models for OHIE

The logistic regression analyses revealed significant differences in the characteristics of users and non-users across the three types of interactions (Table 2).

**Table 2:** Binary logistic regression analyses examining factors associated with online health information exchange with doctors, other healthcare professionals, and peers among older onliners aged 60 + .

Characteristics	Model 1: Digital interactions with doctors (*n* = 972)				Model 2: Digital interactions with other healthcare providers (*n* = 978)				Model 3: Digital interactions with peers (*n* = 976)			
	*p*	OR	95% CI		*p*	OR	95% CI		*p*	OR	95% CI	
			Lower value	Upper value			Lower value	Upper value			Lower value	Upper value
Age, in years	**0**.**012**	1.026	1.006	1.047	0.507	0.993	0.973	1.014	0.156	1.019	0.993	1.047
(registered) Gender Male (Ref.)												
Female	0.364	1.152	0.849	1.565	0.324	0.858	0.633	1.163	0.421	1.180	0.789	1.764
Education Tertiary (Ref.)												
Secondary school	0.090	0.772	0.573	1.041	0.285	0.850	0.631	1.145	0.932	0.982	0.652	1.480
Compulsory	0.927	0.966	0.460	2.030	0.817	1.090	0.525	2.264	**<0**.**001**	4.267	1.977	9.212
Financial situation Easy to very easy (Ref.)												
Rather simple	0.683	1.071	0.771	1.488	0.097	1.317	0.952	1.823	0.142	1.385	0.896	2.141
Very difficult to rather difficult	0.844	0.960	0.642	1.437	0.801	1.053	0.707	1.568	0.144	1.460	0.879	2.424
Living arrangement Not alone (Ref.)												
Living alone	**0**.**001**	0.559	0.394	0.793	0.915	0.982	0.700	1.377	0.649	0.899	0.567	1.425
Support Seeking (range: 1-4)	0.916	1.014	0.783	1.314	0.128	1.223	0.944	1.585	**0**.**001**	1.836	1.281	2.631
Residence location Urban (Ref.)												
Intermediate	0.246	1.226	0.869	1.730	0.215	1.244	0.881	1.757	0.274	1.295	0.815	2.057
Rural	**0**.**023**	0.653	0.453	0.944	0.719	1.067	0.750	1.519	0.274	1.295	0.815	2.059
Internet use (Almost) daily internet use (Ref.)												
Less than (almost) daily	0.244	0.781	0.515	1.184	0.970	0.992	0.655	1.502	0.633	1.134	0.677	1.900
Number of medical treatments Below the mean value (Ref.)												
Above the mean value	**0**.**004**	0.594	0.418	0.843	0.143	0.768	0.540	1.093	0.763	1.075	0.672	1.720
Subjective health status Good to very good (Ref.)												
Very poor to mediocre	0.123	0.750	0.520	1.081	0.714	0.933	0.646	1.349	0.100	0.672	0.419	1.078
Digital competence (DigCompSAT) Advanced (Ref.)												
Intermediate	**<0**.**001**	0.400	0.259	0.616	**0**.**010**	0.579	0.382	0.879	0.268	0.724	0.409	1.282
Basic	**<0**.**001**	0.210	0.121	0.366	**<0**.**001**	0.246	0.142	0.428	**0**.**042**	0.469	0.226	0.974
Low	**<0**.**001**	0.105	0.046	0.236	**<0**.**001**	0.181	0.084	0.392	0.055	0.389	0.148	1.019
Health literacy (HLS19-Q12) Excellent (Ref.)												
Sufficient	0.950	0.985	0.610	1.591	0.792	0.938	0.583	1.510	0.172	0.632	0.328	1.220
Problematic	0.511	1.169	0.734	1.860	0.363	1.238	0.782	1.959	0.400	1.297	0.708	2.377
Deficient	0.865	0.944	0.487	1.830	0.482	1.261	0.661	2.405	0.200	0.535	0.205	1.394

Dependent variable: user OHIE = 1, non-user OHIE = 0. Significant differences in bold. OR, Odds Ratio; Ref.,  reference category; CI,  Confidence Interval. The Hosmer-Lemeshow test indicated no evidence of poor model fit for any of the three models (all *p* > 0.05).

Model 1: Nagelkerkes R^2^ = 0.144; Cox & Snell R^2^ = 0.106; *χ*^2^(19) = 109.243; *p* < 0.001.

Model 2: Nagelkerkes R^2^ = 0.086; Cox & Snell R^2^ = 0.063; *χ*^2^(19) = 63.291; *p* < 0.001.

Model 3: Nagelkerkes R^2^ = 0.093; Cox & Snell R^2^ = 0.053.; *χ*^2^(19) = 53.075; *p* < 0.001.

In the fully adjusted model, older age was associated with slightly higher odds of digital interaction with doctors (OR = 1.026). Participants living alone had lower odds of OHIE with doctors than those living with others (OR = 0.559). Lower odds were also observed among rural residents compared with urban residents (OR = 0.653), and among participants with above-average numbers of medical treatments compared with those with fewer treatments (OR = 0.594).

Moreover, digital competence showed a strong association with OHIE with doctors. Compared to those with advanced digital skills, the odds decreased significantly for intermediate (OR 0.400), basic (OR 0.210), and low competence (OR 0.105) to engage in OHIE with doctors.

For digital interactions with healthcare providers, only digital competence was associated with OHIE in the fully adjusted model. Participants with intermediate (OR 0.579), basic (OR 0.246), and low (OR 0.181) digital competence were significantly less likely to engage with other healthcare providers online. No other sociodemographic, health-related, or contextual variables showed statistically significant associations in this model.

In contrast to professional OHIE, peer-to-peer OHIE was associated with a different set of factors. Participants with compulsory education (i.e., nine years of school in Switzerland) had significantly higher odds of engaging in OHIE with peers than those with tertiary education (OR 4.267). Moreover, individual tendencies to seek social support are particularly noteworthy: people with a stronger tendency to seek support from others in emotionally or practically challenging situations are significantly more likely to engage in OHIE with peers (OR 1.836). Digital competence showed a limited association in this context: only participants with basic digital skills were significantly less likely to interact with peers compared to those with advanced competence (OR 0.469). Other factors did not show any statistical significance in the model. However, given the relatively small number of peer-based OHIE users (*n* = 174), estimates from this model should be interpreted with caution.

### 3.3 Barriers to OHIE among non-users

The most frequently mentioned barrier by non-users of OHIE (see Figure 1) was concerns about the credibility of the information (47%), followed by general distrust of digital offerings (37%). A significant share of respondents cited the prevalence of dubious or untrustworthy sources (35%) and a lack of experience with digital tools (31%). Additional barriers included the absence of social interaction during the information-seeking process (19%) and the use of technical or complex language (14%) in digital health content. Less commonly reported, but still noteworthy, were previous negative experiences (5%), lack of support (4%), physical limitations (2%), and limited access to digital technologies (2%). These results highlight that, beyond structural and technical obstacles, issues of trust and digital literacy represent major barriers to the engagement of older adults with online health information.

**Figure 1:**
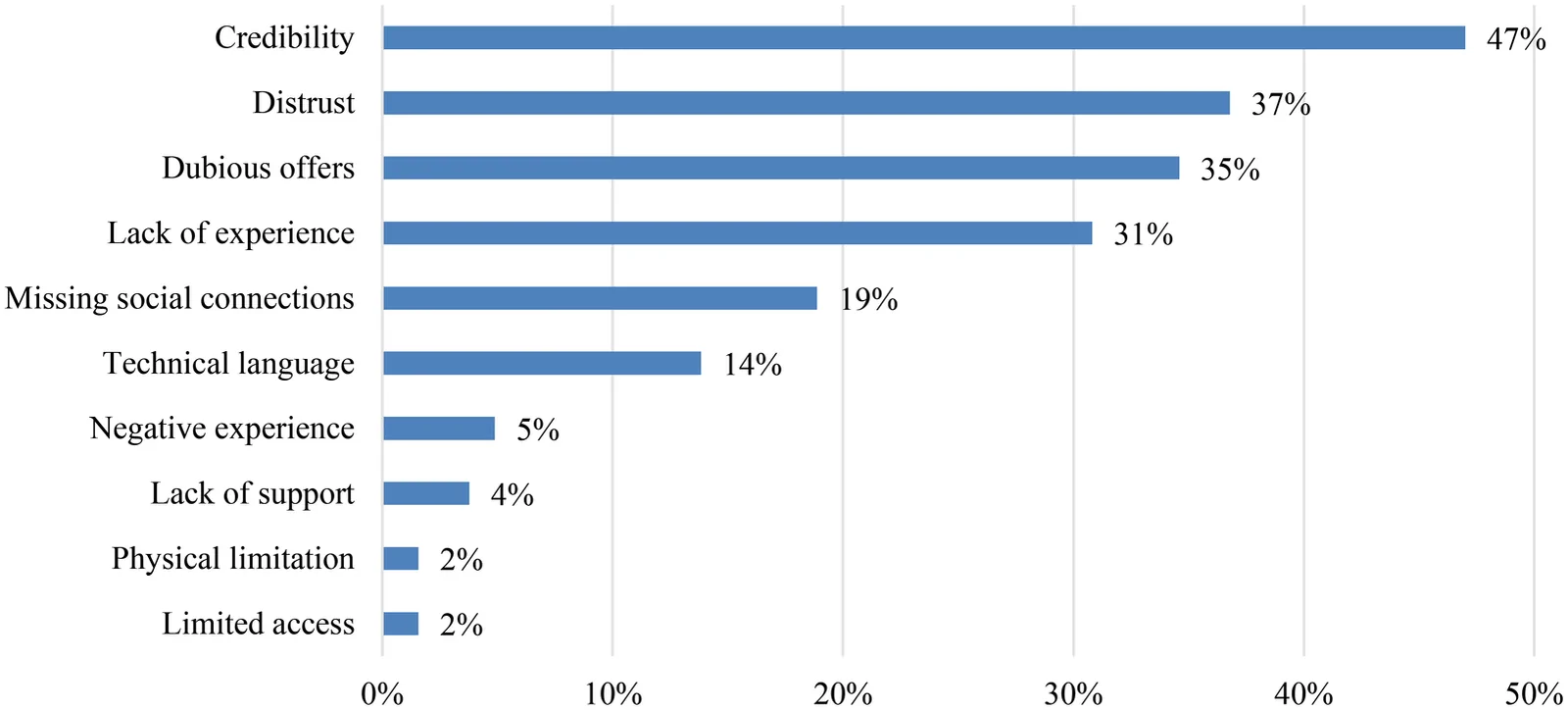
Barriers of all non-users of OHIE (*n* = 660).

## 4 Discussion

### 4.1 Discussion of the findings

This study examined how older adults in Switzerland engage in OHIE with doctors, other health care providers, and peers. Drawing on the framework for digital health equity (16), the findings reveal that OHIE in later life is not a uniform practice but differs depending on the interaction context and is shaped by determinants at the societal, community, individual, and interpersonal levels.

In the Swiss context, data from the last national Health Literacy Survey (26), collected in 2020, indicate that 29% of the general population aged 18 years and over reported using digital services to interact with health care providers, such as physicians or pharmacists. In comparison, the present study found that 36% of adults aged 60 years and over reported using digital services to interact with doctors, and 32% reported doing so with other health care providers. At first glance, this suggests a higher proportion among older adults; however, this comparison should be interpreted with caution, as the present sample included only Internet users and our data were collected four years later; moreover, older adults may have greater health care needs. Nonetheless, the findings indicate that digital interaction with health care professionals is already relatively common among older adults in Switzerland while also pointing to the continued potential for more frequent use of digitally supported health communication.

A central finding is that OHIE with doctors and other health care providers was more common than peer-based OHIE. This suggests that older adults primarily use digital health communication as a channel for formal, expert-oriented exchange rather than for mutual support or experience sharing. This pattern is consistent with the assumption that professional OHIE is closely related to institutional health care structures and perceived medical authority. In contrast, peer-based OHIE was the least common form of exchange, which may reflect concerns regarding privacy, credibility, or the legitimacy of experiential knowledge. It may also indicate that older adults prefer peer exchange in offline settings or through established personal networks rather than through digital environments (27).

Further, the regression results reveal that different forms of OHIE are associated with different determinants. Digital competence was the most consistent correlate across the models, particularly for professional OHIE. Compared with older adults with advanced digital competence, those with low, basic, or intermediate competence had substantially lower likelihood of engaging in digital interactions with doctors or other health care providers. This finding supports previous research that emphasizes digital skills as a central prerequisite for digital health engagement in later life (15, 17). From the perspective of digital health equity, this emphasizes the importance of individual-level resources: even when digital health services are available, older adults may be excluded from their use if they lack the necessary skills, confidence, or practical experience to navigate them.

For OHIE with doctors, additional community- and household-related factors were relevant. Rural residents had lower odds of digital interaction with doctors than urban residents, thus suggesting that local opportunity structures may be associated with differences in access to or familiarity with digitally supported health care communication. This finding is in line with the framework's emphasis on community-level determinants and indicates that digital health engagement is not only a matter of individual competence but also embedded in local health care infrastructures. Moreover, older adults living alone had lower odds of OHIE with doctors than those living with others. Although bivariate results revealed higher use among those not living alone, the adjusted model suggests that household context may be significant independently of other characteristics. One possible interpretation is that cohabiting individuals may benefit from informal support when accessing or utilizing digital health services (28).

The negative association between medical treatments within the last 12 months and OHIE with doctors was unexpected. It could be assumed that greater health care use would increase the needs or opportunities for digital interaction. However, this finding may indicate that older adults with more frequent medical treatments rely more strongly on in-person communication or have more complex health needs that are less easily addressed through digital channels. It may also reflect that digital services are not yet systematically integrated into all areas of routine care. Thus, greater health care embeddedness does not automatically translate into higher digital engagement.

Further, peer-based OHIE followed a different pattern. In contrast to professional OHIE, peer-based OHIE was not primarily associated with community-level factors or general health care use but with education and seeking social support. Older adults with compulsory education had higher odds of engaging in peer-based OHIE than those with tertiary education. This finding contrasts with common assumptions that lower education is generally associated with lower digital health participation (29–31). One possible explanation is that peer-based online exchange may offer a lower-threshold form of health-related support for individuals who may feel less informed by formal or expert-centered information environments. At the same time, this result should be interpreted carefully, as peer-based digital spaces could also increase exposure to misinformation or unreliable information (3).

The strong association between social support-seeking and peer-based OHIE further suggests that interpersonal motivations play a central role in this form of exchange. Older adults who are more inclined to seek support from others were more likely to engage with peers online. This finding aligns with the interpersonal level of the digital health equity framework (16) and indicates that peer-based OHIE does not only depend on digital access or competence but also reflects social needs, help-seeking orientations, and preferences for experiential exchange. Therefore, peer-based digital environments could serve as important spaces for emotional support, shared experience, and social connection, particularly for individuals who actively seek interpersonal resources.

Health literacy was not significantly associated with any form of OHIE in the regression models. This finding does not necessarily imply that health literacy is irrelevant for OHIE. Rather, it suggests that, once digital competence is considered, health literacy may play a less direct role in explaining whether older adults use digital channels for health-related exchange. One possible, speculative explanation is that digital competence captures more immediate practical requirements for OHIE, such as utilizing platforms, navigating interfaces, and managing digital communication. Older adults with sufficient health literacy may still refrain from engaging in OHIE if they lack digital skills, while those with higher digital competence may be better able to compensate for limitations in health literacy through search functions, platform design, or interactive support. However, this interpretation was not tested via mediation analysis in the present study and should be examined directly in future research. This highlights the need to distinguish between understanding health information and being able to access, evaluate, and exchange such information in digital environments.

Furthermore, the descriptive findings on nonuse barriers indicate the societal level of digital health equity. Concerns regarding credibility, general distrust of digital offerings, and the presence of dubious or untrustworthy sources were among the most frequently reported barriers. These findings indicate that nonuse cannot be explained by digital access or skills alone. Trust, perceived legitimacy, and confidence in digital health environments appear to be relevant factors associated with OHIE among older adults (12, 28). Therefore, efforts to promote inclusive digital health communication should not solely focus on improving digital competence but also on strengthening the usability, credibility, transparency, and trustworthiness of digital health services.

Finally, although AI-mediated health information was not examined directly in this study, its increasing use may intensify the need for digital judgment among older adults (32). Generative AI can produce highly plausible health information while making credibility, verification, and appropriate trust more difficult to assess, highlighting the importance of these competencies in future digital health environments (33).

### 4.2 Limitations

While the findings offer important insights, several limitations may affect their generalizability. First, the cross-sectional design limits our ability to draw causal inferences about the relationships between sociodemographic, behavioral, competence-related factors, and OHIE use. Second, the study relies on self-reported data, which may be subject to recall bias and social desirability bias. Participants may underreport or overreport their use of digital health tools or their competencies, particularly in complex areas such as digital competence and health literacy. Third, a stratified random sampling approach was used with the aim of supporting demographic and regional representativeness at the sampling stage; however, given the net response rate of 16.6%, the representativeness of the final analytic sample cannot be assumed and should be interpreted with caution. Additionally, the survey was administered via both CAWI and PAPI modes, and differences between these modes may have influenced self-reports of digital competence and OHIE use; we did not formally test for mode effects, which represents a further limitation. Forth, the findings are embedded in a specific national and institutional context. Features of the Swiss healthcare system, regional variation in healthcare access, digital infrastructure, and sociocultural diversity may shape digital health engagement in ways that differ from other countries. Fifth, the multilingual administration of the questionnaire represents an additional limitation. Although standardized translation procedures were applied to ensure conceptual equivalence across the German, French, and Italian versions, subtle differences in wording, connotations, or cultural interpretation cannot be fully ruled out. Finally, no sampling weights were applied, and no formal non-response analysis or comparison of respondents across the CAWI and PAPI survey modes was conducted. Therefore, residual selection bias related to response mode or non-response cannot be excluded.

## 5 Conclusion

This study shows that, although digital health communication is becoming more popular among older adults, they engage with it in different ways. OHIE with professionals was associated with digital competence, while peer-based exchange was associated with social motivations and lower educational levels. These findings represent meaningful associations that should be confirmed in larger, longitudinal studies with more representative samples. Furthermore, because the analytic sample was restricted to internet users, these findings cannot be generalized to older adults who do not use the internet. However, our findings highlight the importance of interventions that not only aim to strengthen digital competence but also foster trust, usability, and digital judgment as digital health tools become more complex and increasingly integrated into everyday life. Future research should also adopt longitudinal designs to examine changes in OHIE behaviors over time.

## Data Availability

The raw data supporting the conclusions of this article will be made available by the authors, without undue reservation.
